# Downregulation of enhancer RNA EMX2OS is associated with poor prognosis in kidney renal clear cell carcinoma

**DOI:** 10.18632/aging.202151

**Published:** 2020-11-25

**Authors:** Huiming Jiang, Haibin Chen, Pei Wan, Shengda Song, Nanhui Chen

**Affiliations:** 1 Department of Urology, Meizhou People’s Hospital, Guangdong Provincial Key Laboratory of Precision Medicine and Clinical Translational Research of Hakka Population, Meizhou 514031, Guangdong Province, P.R. China; 2 Department of Histology and Embryology, Shantou University Medical College, Shantou 515041, Guangdong Province, P.R. China

**Keywords:** kidney renal clear cell carcinoma, eRNA, EMX2OS, prognosis

## Abstract

Enhancer RNAs are a subclass of long non-coding RNAs transcribed from enhancer regions that play an important role in the transcriptional regulation of genes. However, their role in kidney renal clear cell carcinoma (KIRC) is largely unknown. Herein, we identified the key enhancer RNAs in KIRC via an integrated data analysis method. Gene expression profiles and clinical data of KIRC and 32 other cancer types were acquired using the University of California Santa Cruz Xena platform. Reported enhancer RNAs and genes regulated by them were selected as putative enhancer RNA-target pairs. Kaplan-Meier survival and correlation analyses were performed to identify the key enhancer RNAs. Finally, EMX2OS was identified as the enhancer RNA most associated with survival, with *EMX2* as its target. EMX2OS downregulation was significantly associated with higher histological grade, advanced stage, and poorer prognosis. The results were validated in pan-cancer data from The Cancer Genome Atlas and RT-qPCR analysis of 12 pairs of KIRC and normal real-world samples. Functional enrichment analysis indicated that several metabolism-associated signaling pathways were enriched. This study demonstrated that EMX2OS is a key metabolism-associated enhancer RNA in KIRC with a favorable impact on survival and may be a novel therapeutic target in KIRC.

## INTRODUCTION

Kidney renal clear cell carcinoma (KIRC) is the most common histological type of renal cell carcinoma and is associated with the highest metastasis and mortality rates [1]. The number of new cases of KIRC was 73,820 in the United States, and it had caused approximately 14,770 deaths based on cancer statistics data in 2019 [2]. Contrast-enhanced, triple-phase helical computed tomography (CT) and surgery are the most important diagnostic and treatment methods, respectively [3]. Although the treatment for KIRC has improved significantly, its mortality rate is still increasing [2]. Up to 50% of patients who undergo nephrectomy will progress to distant metastasis, and the 5-year survival rate of patients with metastasis is below 12% [4]. The TNM stage remains the most important prognostic predictor for KIRC, but the survival rate can differ greatly for patients even with the same stage because of tumor heterogeneity [5]. Therefore, it is essential to identify effective prognostic biomarkers to identify high-risk patients with poor survival.

Enhancer RNAs (eRNAs) are a subclass of long non-coding RNAs transcribed from enhancer regions. eRNAs are known to promote the formation of enhancer–promoter loops and play diverse and emerging roles in the regulation of gene expression and cell fate [6–8]. In addition, increasing evidence suggests that eRNAs are closely associated with carcinogenesis [9–13]. However, few studies have investigated the role of eRNAs in KIRC. In this study, we explored and identified the eRNAs associated with survival in patients with KIRC. We found that eRNA EMX2OS, located in the tissue-specific enhancer of a tumor suppressor gene EMX2 [14–16], was positively correlated with favorable clinicopathological characteristics and was significantly related to overall survival (OS) in patients with KIRC.

## RESULTS

### Putative prognostic eRNAs in KIRC

After matching the patients’ clinical information and gene expression data, 535 patients with KIRC were enrolled in the study. Among them, 531 patients had information regarding survival. The patients’ clinicopathological features are summarized in Table 1. A total of 2695 eRNAs, which were annotated using the Encyclopedia of DNA Elements database and derived from tissue-specific enhancers, as well as 2303 predicted target genes, have been identified previously using the PreSTIGE algorithm [17]. We used these eRNA-target pairs to identify potential key eRNAs in KIRC. Finally, 16 eRNA-target pairs were identified with certain conditions (Kaplan-Meier log rank of p < 0.001 and correlation coefficient of r > 0.6 and p < 0.001) (Table 2).

**Table 1:** Correlations between the expression of EMX2OS/*EMX2* and clinicopathologic characteristics in KIRC.

**Characteristic**	**n (%)**	**EMX2OS expression (%)**		*p*-value*	***EMX2* expression (%)**		*p*-value*
		**High**	**Low**		**High**	**Low**	
Total	535 (100)	267 (49.91)	268 (50.09)		267 (49.91)	268 (50.09)	
Age				0.897			0.195
≤ 60 years	267 (49.91)	132 (49.4)	135 (50.4)		141 (52.8)	126 (47.0)	
> 60 years	268 (50.09)	135 (50.6)	133 (49.6)		126 (47.2)	142 (53.0)	
Gender				0.001			0.029
Female	186 (34.77)	111 (41.6)	75 (28.0)		105 (39.3)	81 (30.2)	
Male	349 (65.23)	156 (58.4)	193 (72.0)		162 (60.7)	187 (69.8)	
Cancer status				< 0.001			< 0.001
Tumor free	336 (62.8)	186 (69.7)	150 (56.0)		186 (69.7)	150 (56.0)	
With tumor	148 (27.66)	47 (17.6)	101 (37.7)		48 (18.0)	100 (37.3)	
Unknow	51 (9.53)	34 (12.7)	17 (6.3)		33 (12.4)	18 (6.7)	
Race				0.021			0.015
White	463 (86.54)	222 (83.1)	241 (89.9)		222 (83.1)	241 (89.9)	
Asian	8 (1.5)	5 (1.9)	3 (1.1)		4 (1.5)	4 (1.5)	
Black	57 (10.65)	38 (14.2)	19 (7.1)		39 (14.6)	18 (6.7)	
Unknow	7 (1.31)	2 (0.7)	5 (1.9)		2 (0.7)	5 (1.9)	
Grade				< 0.001			< 0.001
G1	14 (2.62)	13 (4.9)	1 (0.4)		13 (4.9)	1 (0.4)	
G2	231 (43.18)	137 (51.3)	94 (35.1)		142 (53.2)	89 (33.2)	
G3	207 (38.69)	100 (37.5)	107 (39.9)		93 (34.8)	114 (42.5)	
G4	75 (14.02)	14 (5.2)	61 (22.8)		17 (6.4)	58 (21.6)	
Unknow	8 (1.5)	3 (1.1)	5 (1.9)		2 (0.7)	6 (2.2)	
T stage				< 0.001			< 0.001
T1	275 (51.4)	164 (61.4)	111 (41.4)		166 (62.2)	109 (40.7)	
T2	70 (13.08)	32 (12.0)	38 (14.2)		31 (11.6)	39 (14.6)	
T3	179 (33.46)	70 (26.2)	109 (40.7)		69 (25.8)	110 (41.0)	
T4	11 (2.06)	1 (0.4)	10 (3.7)		1 (0.4)	10 (3.7)	
N stage				0.008			< 0.001
N0	240 (44.86)	112 (41.9)	128 (47.8)		115 (43.1)	125 (46.6)	
N1	16 (2.99)	2 (0.7)	14 (5.2)		0 (0.0)	16 (6.0)	
Unknow	279 (52.15)	153 (57.3)	126 (47.0)		152 (56.9)	127 (47.4)	
M stage				0.001			< 0.001
M0	424 (79.25)	219 (82.0)	205 (76.5)		219 (82.0)	205 (76.5)	
M1	78 (14.58)	24 (9.0)	54 (20.1)		23 (8.6)	55 (20.5)	
Unknow	33 (6.17)	24 (9.0)	9 (3.4)		25 (9.4)	8 (3.0)	
AJCC stage				< 0.001			< 0.001
Stage I	269 (50.28)	163 (61.0)	106 (39.6)		164 (61.4)	105 (39.2)	
Stage II	58 (10.84)	28 (10.5)	30 (11.2)		26 (9.7)	32 (11.9)	
Stage III	123 (22.99)	51 (19.1)	72 (26.9)		51 (19.1)	72 (26.9)	
Stage IV	82 (15.33)	24 (9.0)	58 (21.6)		25 (9.4)	57 (21.3)	
Unknow	3 (0.56)	1 (0.4)	2 (0.7)		1 (0.4)	2 (0.7)	

KIRC, Kidney renal clear cell carcinoma; AJCC, American Joint Committee on Cancer.

*Patients with the missing details were excluded when calculating the *p* value of the corresponding characteristic.

**Table 2:** Survival-associated eRNAs and their predicted target.

**eRNA**	**Log-rank test p-value**	**Predicted target**	**correlation coefficient r**	**Spearman p-value**
EMX2OS	3.91E-07	EMX2	0.809	<0.001
CYP1B1-AS1	1.94E-06	CYP1B1	0.669	<0.001
CCDC18-AS1	4.18E-06	CCDC18	0.748	<0.001
LINC00323	6.03E-05	BACE2	0.765	<0.001
JPX	8.10E-05	XIST	0.679	<0.001
STX4	8.13E-05	FBXL19	0.623	<0.001
STX4	8.13E-05	HSD3B7	0.625	<0.001
STX4	8.13E-05	PRSS53	0.651	<0.001
SSPO	0.000105	ZNF862	0.678	<0.001
LINC00937	0.000228	POU5F1P3	0.643	<0.001
BAALC-AS1	0.000329	FZD6	0.672	<0.001
HOTAIR	0.000461	HOXC11	0.688	<0.001
WDFY3-AS2	0.000473	WDFY3	0.641	<0.001
LINC00671	0.000583	G6PC	0.705	<0.001
AFG3L1P	0.000591	MC1R	0.786	<0.001
APCDD1L-DT	0.000724	APCDD1L	0.855	<0.001

### EMX2OS is a key eRNA in KIRC

**Figure 1:**
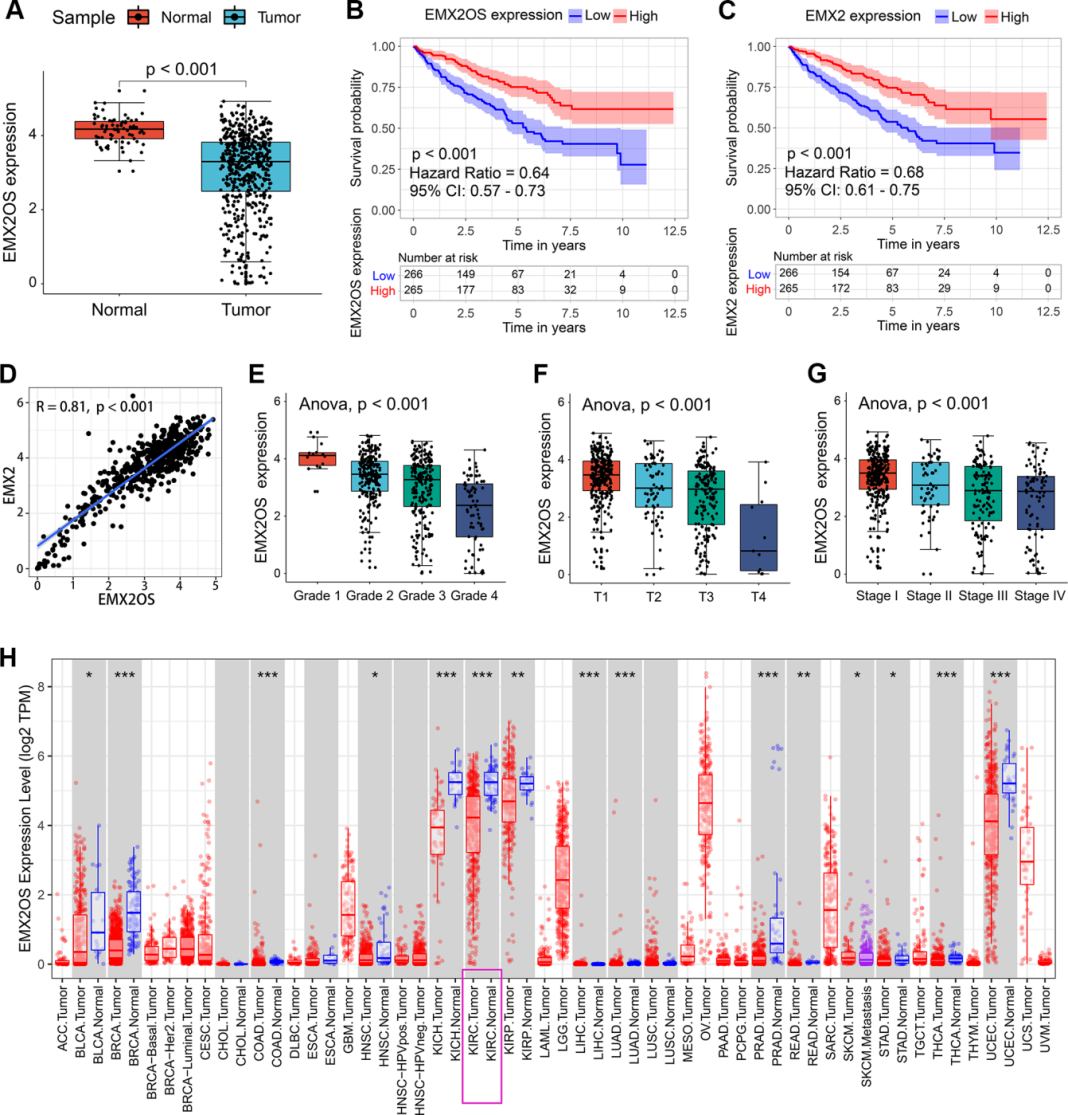
**Association between EMX2OS expression and the key clinicopathological characteristics of KIRC.** (**A**) EMX2OS expression was significantly lower in KIRC tissue samples than in normal tissue samples. (**B**) EMX2OS downregulation was significantly correlated with poorer overall survival in KIRC. (**C**) *EMX2* downregulation was significantly correlated with poorer overall survival in KIRC. (**D**) Correlation analysis between EMX2OS and its predicted target *EMX2*. (**E**) EMX2OS expression significantly decreased with increasing histological grade. (**F**) EMX2OS expression significantly decreased with increasing tumor size (T stage). (**G**) EMX2OS expression significantly decreased with advanced AJCC stages. (**H**) EMX2OS expression in pan-cancer from TIMER database (https://cistrome.shinyapps.io/timer/). KIRC, kidney renal clear cell carcinoma; AJCC, American Joint Committee on Cancer.

As shown in Table 2, EMX2OS was the putative eRNA most associated with survival, and it showed an obviously positive correlation with its predicted target EMX2. Thus, we performed further analysis for EMX2OS. Based on the expression data of 535 KIRC samples and 72 normal kidney samples, we found that EMX2OS expression was significantly lower in tumor tissues than in normal tissues (p < 0.001) (Figure 1A). According to the median expression level of EMX2OS, 531 patients with KIRC with survival information were divided into the high-EMX2OS and low-EMX2OS expression groups. Kaplan–Meier survival analysis demonstrated that EMX2OS downregulation was significantly associated with poorer OS (hazard ratio [HR] = 0.64, p < 0.001, Figure 1B). Lower EMX2 expression was also related to poorer OS (HR = 0.68, p < 0.001, Figure 1C). Furthermore, there was a strong co-expression relationship between EMX2OS and its predicted target EMX2 (correlation coefficient r = 0.81; p < 0.001) (Figure 1D). We next investigated the relationship between EMX2OS expression and the clinicopathological characteristics of KIRC. We found that the EMX2OS expression level was significantly related to several clinicopathological features of KIRC, including gender (p = 0.001), cancer status (p < 0.001), race (p = 0.021), histological grade (p < 0.001), tumor size (T stage, p < 0.001), lymph node stage (N stage, p = 0.008), distant metastasis (M stage, p = 0.001), and American Joint Committee on Cancer (AJCC) stage (p < 0.001) (Table 1). Moreover, EMX2 expression showed a similar relationship with the clinicopathological characteristics of KIRC (Table 1). Further analysis showed that EMX2OS expression was negatively correlated with histological grade (p < 0.001), T stage (p < 0.001), and AJCC stage (p < 0.001) (Figure 1E–1G). To validate the results, we explored the differential expression and prognostic value of EMX2OS and its correlation with EMX2 in pan-cancer data (32 other cancer types) from The Cancer Genome Atlas (TCGA) as an internal validation. Interestingly, EMX2OS expression was significantly lower in many other types of cancers, including breast invasive carcinoma, colon adenocarcinoma, kidney chromophobe, and liver hepatocellular carcinoma, than in normal tissues (Figure 1H). EMX2OS also played a prognostic role in adrenocortical carcinoma, cervical squamous cell carcinoma and endocervical adenocarcinoma, stomach adenocarcinoma, and uveal melanoma (Table 3). Of interest, EMX2OS and EMX2 showed significant correlations in all cancers, except cholangiocarcinoma (Table 3). These results suggest that EMX2OS functions as a tumor suppressor in KIRC.

**Table 3:** Kaplan-Meier survival analysis and correlations analysis for EMX2OS and *EMX2* in pan-cancer (33 types of cancer from TCGA).

**Abbreviation**	**Detail**	**Log-rank test p-value**	**Correlation coefficient r**	**Spearman p-value**
ACC	Adrenocortical carcinoma	0.002	0.844	< 0.001
BLCA	Bladder Urothelial Carcinoma	0.083	0.952	< 0.001
BRCA	Breast invasive carcinoma	0.989	0.917	< 0.001
CESC	Cervical squamous cell carcinoma and endocervical adenocarcinoma	0.020	0.954	< 0.001
CHOL	Cholangio carcinoma	0.061	0.311	0.065
COAD	Colon adenocarcinoma	0.783	0.649	< 0.001
DLBC	Lymphoid Neoplasm Diffuse Large B-cell Lymphoma	0.744	0.713	< 0.001
ESCA	Esophageal carcinoma	0.872	0.710	< 0.001
GBM	Glioblastoma multiforme	0.226	0.924	< 0.001
HNSC	Head and Neck squamous cell carcinoma	0.498	0.862	< 0.001
KICH	Kidney Chromophobe	0.121	0.792	< 0.001
KIRC	Kidney renal clear cell carcinoma	0.000	0.809	< 0.001
KIRP	Kidney renal papillary cell carcinoma	0.068	0.787	< 0.001
LAML	Acute Myeloid Leukemia	0.762	0.594	< 0.001
LGG	Brain Lower Grade Glioma	0.172	0.919	< 0.001
LIHC	Liver hepatocellular carcinoma	0.979	0.418	< 0.001
LUAD	Lung adenocarcinoma	0.359	0.514	< 0.001
LUSC	Lung squamous cell carcinoma	0.886	0.694	< 0.001
MESO	Mesothelioma	0.838	0.928	< 0.001
OV	Ovarian serous cystadenocarcinoma	0.859	0.799	< 0.001
PAAD	Pancreatic adenocarcinoma	0.634	0.793	< 0.001
PCPG	Pheochromocytoma and Paraganglioma	0.504	0.835	< 0.001
PRAD	Prostate adenocarcinoma	0.382	0.847	< 0.001
READ	Rectum adenocarcinoma	0.829	0.703	< 0.001
SARC	Sarcoma	0.986	0.938	< 0.001
SKCM	Skin Cutaneous Melanoma	0.126	0.896	< 0.001
STAD	Stomach adenocarcinoma	0.002	0.762	< 0.001
TGCT	Testicular Germ Cell Tumors	0.440	0.891	< 0.001
THCA	Thyroid carcinoma	0.356	0.862	< 0.001
THYM	Thymoma	0.141	0.746	< 0.001
UCEC	Uterine Corpus Endometrial Carcinoma	0.677	0.860	< 0.001
UCS	Uterine Carcinosarcoma	0.529	0.908	< 0.001
UVM	Uveal Melanoma	0.013	0.808	< 0.001

### Independent prognostic value of EMX2OS in KIRC

**Figure 2:**
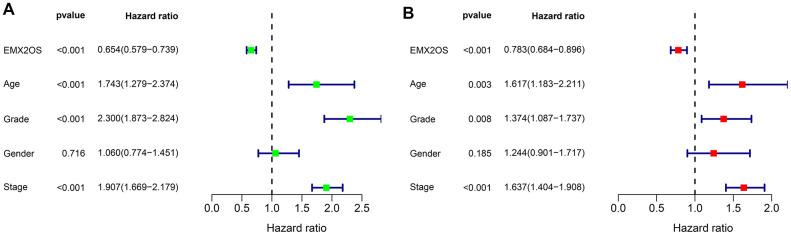
**Forest plot of Cox regression analysis in KIRC.** (**A**) Forest plot of univariate Cox regression analysis. (**B**) Forest plot of multivariate Cox regression analysis.

**Figure 3:**
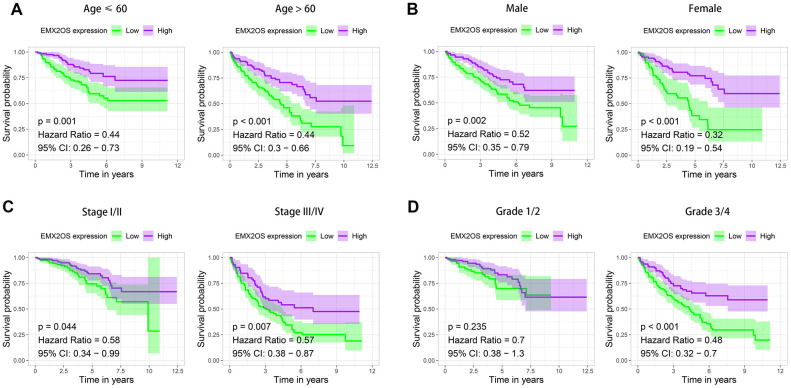
**Kaplan–Meier survival analysis of EMX2OS expression according to age, gender, stage, and grade stratification.** (**A**) Age ≤ 60 years and age > 60 years. (**B**) Male and female. (**C**) Stage I/II and stage III/IV. (**D**) Grade 1/2 and grade 3/4.

As Kaplan–Meier survival analysis showed that patients with different EMX2OS expressions had significant different OS, we further explored whether EMX2OS had an independent prognostic value in KIRC using univariate and multivariate Cox regression analyses. Because information on the M stage, N stage, T stage, cancer status, and race were missing or were unevenly distributed in most cases, these data were excluded. Finally, 520 patients with information on age, gender, histological grade, AJCC stage, and EMX2OS expression were included in the Cox regression analysis. We found that there was a significantly prognostic difference between the high-EMX2OS and low-EMX2OS expression groups in both univariate (HR, 0.654; 95%CI, 0.579 - 0.739; p < 0.001) and multivariate (HR, 0.783; 95%CI, 0.684 - 0.896; p < 0.001) Cox regression analysis (Figure 2A, 2B). EMX2OS expression, age, histological grade, and AJCC stage had independent prognostic values in KIRC. Additionally, the stratified analysis revealed that the patients in the low-EMX2OS expression group had significantly poorer OS than those in the high-EMX2OS expression group regarding age ≤ 60 years (p = 0.001), age > 60 years (p < 0.001), male sex (p = 0.002), female sex (p < 0.001), AJCC stage I/II (p = 0.044), AJCC stage III/IV (p = 0.007), and histological grade ¾ (p < 0.001) but not histological grade 1/2 (p = 0.235) (Figure 3A–3D).

### Determination of EMX2OS and EMX2 levels using RT-qPCR

**Figure 4:**
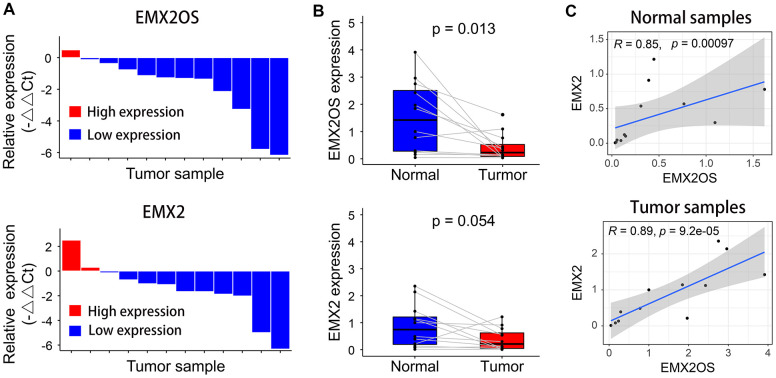
**Relative expression levels of EMX2OS and *EMX2* in 12 pairs of KIRC and normal samples measured using RT-qPCR.** (**A**) EMX2OS and *EMX2* were downregulated in 11 of 12 and 10 of 12 KIRC samples, respectively. (**B**) Compared with than in normal samples, EMX2OS was significantly downregulated in tumor samples, while no significant difference was found for *EMX2*. (**C**) There was a significant positive correlation between EMX2OS and *EMX2* in both normal and tumor samples.

Quantitative reverse transcription polymerase chain reaction (RT-qPCR) was performed to measure the relative expression of EMX2OS and EMX2 in 12 patients with KIRC. Compared with the matched tumor-free samples, EMX2OS and EMX2 expression levels were downregulated in 91.7% (11 of 12) and 83.3% (10 of 12) of KIRC samples, respectively (Figure 4A). However, compared with normal samples, only EMX2OS was significantly downregulated in KIRC samples (p = 0.013), although it was nearly significantly different for EMX2 (p = 0.054), possibly because of the small sample size (Figure 4B). Additionally, there was an obviously significant positive correlation between EMX2OS and EMX2 in both normal (r = 0.85; p < 0.001) and tumor samples (r = 0.89; p < 0.001) (Figure 4C).

### Functional enrichment analysis

**Figure 5:**
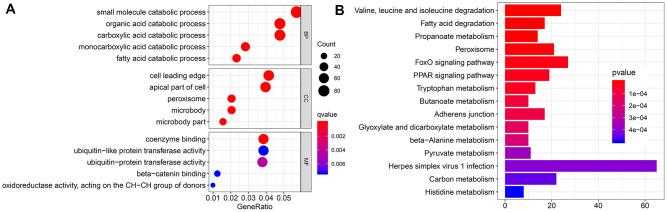
**Functional enrichment analysis.** (**A**) Bubble chart shows the result of Gene Ontology (GO) analysis. (**B**) Bar chart shows the top 15 pathways enriched by Kyoto Encyclopedia of Genes and Genomes (KEGG).

To further investigate the function of EMX2OS, we identified 2124 significantly co-expressed genes with EMX2OS in KIRC (r > 0.4; p < 0.001) (Supplementary Table 1). Gene Ontology (GO) functional enrichment analysis showed that several catabolic processes, including small molecule, carboxylic acid, and fatty acid catabolic processes, were significantly enriched (Figure 5A). Kyoto Encyclopedia of Genes and Genomes (KEGG) analysis revealed that the metabolism of several important energy substances, including fatty acid, pyruvate, and tryptophan, was enriched. Moreover, FOXO and PPAR signaling pathways were also enriched (Figure 5B).

## DISCUSSION

The treatment for KIRC remains a challenge across the world because of its substantial morbidity and mortality [18]. Thus, identifying novel markers for prognosis and therapy is needed to improve the survival of patients with KIRC. Recently, with rapid advanced in high-throughput sequencing technology and bioinformatics, more novel biomarkers have been identified. One of the most surprising discoveries is eRNAs. Accumulation evidence has shown that the dysregulation of eRNAs is closely associated with various human diseases and that eRNAs are a new therapeutic target [19–22].

eRNAs are transcribed from putative enhancer regions distinguished by high levels of histone H3 lysine 27 acetylation and histone H3 lysine 4 monomethylation and low levels of histone H3 lysine 4 trimethylation. PreSTIGE is a recently developed algorithm to predict tissue-specific enhancers and their targets based on the H3K4me1 marker and tissue-specific expression of mRNAs [23]. Using this enhancer prediction approach, 2695 eRNAs and their predicted targets were identified in a previous study [17]. As the role of eRNAs had been rarely studied in KIRC, we used these 2695 eRNA– target pairs as candidates to identify key eRNAs in KIRC. Using Kaplan–Meier survival and correlation analyses, EMX2OS was found to be the most survival-associated eRNA with high correlation with its predicted target EMX2.

EMX2OS is a long non-coding RNA, an antisense transcript from EMX2 [24]. EMX2 encodes a homeobox protein and is a vital gene that promotes the formation of the urogenital and central nervous systems during embryonic development [25, 26]. In cancer, EMX2 usually acts as a tumor suppressor [15, 16]. Although rarely reported, we speculate that EMX2OS also serves as a tumor suppressor because of its strong co-expression relationship with EMX2. In this study, we found that EMX2OS was significantly downregulated in KIRC tissues compared with that in normal kidney tissues. EMX2OS downregulation was significantly associated with unfavorable clinicopathological features such as higher histological grade, larger tumor size, lymph node metastasis, distant metastasis, and advanced AJCC stage. Kaplan–Meier survival analysis showed that downregulated EMX2OS expression was significantly associated with poorer OS. Furthermore, multivariate Cox regression analysis confirmed that EMX2OS played an independent prognostic role in KIRC. Additionally, patients with lower EMX2OS expression had significantly poorer OS than patients with higher EMX2OS expression in stratified analysis according to age, gender, and AJCC stage. However, a significant difference in survival was found in patients with histological grade 3/4 but not in those with histological grade 1/2. A possible reason for this difference is that the tumors with low histological grades were relatively less malignant.

To validate the aforementioned results, we used data of 32 other types of cancer from TCGA as internal validation and RT-qPCR analysis of 12 pairs of KIRC and normal real-world samples as external validation. Pan-cancer analysis showed that significant expression and survival differences of EMX2OS existed in several types of cancers. The RT-qPCR results further confirmed the downregulation of EMX2OS in KIRC. All results were consistent and suggested that EMX2OS serves as a tumor suppressor in KIRC.

GO and KEGG functional enrichment analyses provided some clues on how EMX2OS influences patients’ survival. These analyses showed that the metabolism of several energy-involved substances, such as fatty acid and pyruvate, were enriched, which suggests that EMX2OS influences the energy metabolism of the tumor. In the KEGG pathway analysis, FOXO and PPAR signaling pathways were also enriched. The FOXO signaling pathway plays an important role in the regulation of metabolic homeostasis and suppression of tumor growth [27–29]. PPARs are nuclear receptors that regulate cellular and whole-body energy homeostasis during carbohydrate and lipid metabolism, cell growth, and cancer development [30]. Alternatively, energy metabolism reprogramming is an emerging hallmark of cancers [31]. Thus, we speculated that EMX2OS may regulate energy metabolism by enriching the FOXO and PPAR signaling pathways to influence the prognosis of KIRC.

Although this study discovered the potential prognostic value of EMX2OS in KIRC for the first time, several limitations need to be considered. First, our data were mainly sourced from TCGA, and most patients were white. Second, the tissue specimens used for RT-qPCR analyses were limited. Therefore, additional data and samples from different races are necessary to confirm the results of this study. Finally, basic experiments are essential to detect the molecular mechanism of EMX2OS in KIRC.

In conclusion, this study demonstrated that EMX2OS is a key survival-associated eRNA in KIRC. With a potential role in energy metabolism, EMX2OS may be a novel therapeutic target in patients with KIRC.

## MATERIALS AND METHODS

### Data extraction and identification of prognostic eRNAs in KIRC

The gene expression profiles and clinical data of KIRC and 32 other types of cancers from TCGA were acquired using the University of California Santa Cruz Xena database (https://xena.ucsc.edu/). We matched the patients’ clinical information and gene expression data. Thus, patients with clinical information and gene expression data were enrolled for further analysis. Next, we obtained a list of eRNAs transcribed from active tissue-specific enhancers and their target predicted using the Predicting Specific Tissue Interactions of Genes and Enhancers (PresSTIGE) method [17, 23]. The association between the level of putative eRNAs and OS of patients with KIRC was investigated using the R packages “survival” and “survminer” (The R Foundation for Statistical Computing, Vienna, Austria). Co-expression analysis was also performed to evaluate the correlation between the level of eRNAs and their predicted targets. Putative eRNAs were considered candidates if they satisfied the following two criteria: significant association with OS (Kaplan–Meier log rank of p < 0.001) and co-expression with the predicted target (r > 0.6 and p < 0.001). Then, we selected the most significant survival-associated eRNA for further analysis. The differential expression between tissues with KIRC and normal tissues was explored. The independent prognostic role of eRNAs in KIRC was investigated. The correlation between the expression level of the selected eRNA and clinicopathological characteristics in KIRC was also assessed. Finally, we validated the results using the pan-cancer data from TCGA (32 other types of cancers) and RT-qPCR results of 12 pairs of KIRC and normal real-world samples as internal and external validations, respectively.

### RT-qPCR

RT-qPCR was used to further validate the RNA-sequencing data obtained from TCGA. We collected KIRC tissues and paired adjacent normal tissues from 12 patients who underwent nephrectomies or partial nephrectomies at Meizhou People’s Hospital between 2019 and 2020. Informed consent was obtained from all patients. We extracted total RNA using the TRIzol™ reagent (Waltham, Massachusetts, USA). The PrimeScript RT reagent kit (Takara Bio, Inc., Dalian, China) was used to synthesize complementary DNAs following the manufacturer’s protocols. The SYBR Green PCR kit (Takara Bio, Inc., Dalian, China) was used to conduct quantitative real-time PCR using the ABI 7500 fluorescent quantitative PCR system (Applied Biosystems Inc., Foster City, CA, USA). We used glyceraldehyde 3-phosphate dehydrogenase (GAPDH) as the internal control. The primer sequences were as follows: EMX2OS (forward: GTGACTTGCACAAGGACACAA; reverse: CCTGTCTGGCCATTCCTCT), EMX2 (forward: CGGCACTCAGCTACGCTAAC; reverse: CAAGTCCGGGTTGGAGTAGAC), and GAPDH (forward: ATGACATCAAGAAGGTGGTG; reverse: CATACCAGGAAATGAGCTTG). The expressions of EMX2OS and EMX2 were measured using 2−ΔΔCt method.

### Functional enrichment analysis

To determine the underlying molecular mechanisms, we explored the co-expressed genes with the selected eRNA (correlation coefficient r > 0.4, p < 0.001). Then, GO and KEGG analyses were performed.

### Statistics

All data processing and statistical analysis were performed using R (version 3.6.1; The R Foundation for Statistical Computing, Vienna, Austria), Strawberry Perl (version 5.30.1.1; http://strawberryperl.com/), and Statistical Package for Social Sciences (version 25.0; IBM, Armonk, New York, USA). Analysis of variance or t-test was used to compare the gene expression level among different subgroups. Spearman’s rank correlation coefficient was used to evaluate the correlation strength. A result was considered statistically significant when the p value was < 0.05, except when stated otherwise.

## Supplementary Material

Supplementary Table 1
